# Reducing alcohol consumption among adolescents: intervention strategies by Chinese healthcare professionals

**DOI:** 10.3389/fpubh.2025.1598803

**Published:** 2025-09-18

**Authors:** Ting Zhou, Yan Wang, Zhijun Xiao, Shaohong Xu, Feng Xu

**Affiliations:** ^1^Sixth People's Hospital South Campus, Shanghai Jiaotong University, Shanghai, China; ^2^Fengxian Hospital, Southern Medical University, Shanghai, China; ^3^Shanghai DeltaHealth Hospital, Shanghai, China

**Keywords:** alcohol consumption, healthcare professionals, health education, intervention, China

## Introduction

Alcohol consumption in China is deeply embedded within the cultural, social, and historical fabric of the nation, serving as a cornerstone of rituals, social cohesion, and philosophical traditions. Its significance transcends mere indulgence, embodying values of hospitality, unity, and respect, which profoundly influence the behavioral development of Chinese adolescents (1, 2). However, alcohol use remains a leading risk factor for global disease burden, contributing to significant health deterioration. Epidemiological studies demonstrate a linear relationship between alcohol consumption and increased all-cause mortality, particularly from cancers, with the optimal level of consumption for minimizing health loss being zero (3). Between 1990 and 2016, alcohol-related fatalities among Chinese males reached 650,822, establishing alcohol as the predominant cause of mortality among young and middle-aged men (3). Given these epidemiological studies data, addressing adolescent alcohol consumption in China demands urgent societal attention and concerted public health efforts.

While alcohol's cultural embeddedness complicates public health responses, epidemiological urgency demands immediate action. This review elaborates on the current status and investigations of adolescent alcohol consumption, explores a critical implementation gap: How can Chinese healthcare professionals develop culturally resonant intervention strategies to reduce adolescent alcohol consumption without alienating deeply rooted social norms? Through rigorous analysis of practice-based constraints, we propose an actionable integration model that bridges health promotion objectives with culturally informed behavioral modification.

## Cultural traditions and social determinants of adolescent drinking

The historical legacy of alcohol in China spans over four millennia, with archaeological evidence tracing fermented beverages to the Neolithic period. Alcohol played a pivotal role in religious rites and ancestral worship, serving as a sacred medium to honor deities and bridge human and divine realms. During the Zhou Dynasty (1046–256 BCE), ceremonial drinking was codified through “wine etiquette,” reflecting its institutionalized role in imperial banquets and diplomatic affairs. Chinese classical poetry romanticizes alcohol as a cultural signifier—from Li Bai's solitary communion (“Amidst flowers, a jug of wine”) to Wang Wei's farewell libations (“one more cup west of Yang Pass”). These texts, canonized in school curricula, implicitly normalize drinking as literary elegance. Through curriculum exposure, adolescents internalize these cultural artifacts, inadvertently perpetuating the romanticization of alcohol in Chinese literary heritage (4).

In contemporary society, alcohol remains integral to social and familial bonds. The act of offering alcohol symbolizes warmth and trust, while refusal may be construed as disrespect, particularly in traditional contexts. Alcohol is indispensable in celebrations such as weddings, reunions, and festivals, where it reinforces communal ties. In business culture, toasting—often involves the emptying of one's glass, which signifies mutual respect and cements professional relationships. Adolescents frequently encounter social pressures to participate in drinking rituals, such as toasting teachers or superiors during graduation or promotion banquets, as a demonstration of respect and deference. Young people have also noticed the effects of alcohol consumption on their performance from their seniors in the workplace and are deeply influenced by it.

Social network is important to firm managers in that it helps to accumulate social capital, build trust and acquire information. An interesting research investigated the impact of Chinese local alcohol drinking culture on CEOs social connections. They found that CEOs whose firms are located in regions with strong alcohol drinking culture have more social connections, both inside and outside the firms. Besides, CEO's inter-firm but not intra-firm social connections established from alcohol drinking can help firms acquire more trade credit (5).The “2023 China Moderate Drinking and Happy Living Blue Book” further demonstrates that nearly 50% of respondents view drinking as a means to enhance social bonds (6).

Cultural festivals further perpetuate alcohol consumption. During the Spring Festival, families share alcohol to invoke prosperity and longevity, while the Dragon Boat Festival features realgar wine as a talisman against malevolent spirits. The Mid-Autumn Festival pairs mooncakes with osmanthus wine to celebrate familial unity. On such occasions, adolescents are often encouraged to toast older adults as a gesture of filial piety. A survey on drinking culture among youth revealed that 43.93% of drinking behaviors occur at home, indicating a direct link between cultural rituals and alcohol consumption (7). A survey evidence indicates that traditional cultural norms constitute a significant determinant influencing adolescents' alcohol consumption behaviors (8). These findings collectively confirm the profound influence of traditional Chinese culture on adolescent drinking behaviors.

Concurrently, evolving sociocultural dynamics have introduced novel determinants of adolescent alcohol use. Emerging evidence highlights the escalating mental health challenges among post-1990s and Generation Z cohorts, characterized by pervasive feelings of existential void, social alienation, and maladaptive coping mechanisms. A cross-sectional study of 1,634 adolescents (aged 18–34 years) revealed that 45.84% of young drinkers initiated alcohol. The earlier the age of first alcohol drinking or the age of first being intoxicated, the greater the likelihood of being a moderate or heavy drinker (9). A nationwide survey of 105,752 Chinese students (aged 9–21 years) revealed significant psychosocial predictors of alcohol use: adolescents with poor classmate relations (AOR = 1.28) or teacher-student relationships (AOR = 1.08) demonstrated elevated drinking risks, particularly among those reporting suicidal ideation (AOR = 1.70–2.08) or higher depressive symptoms (CES-D AOR = 1.08–1.09). Academic underachievers showed 50% increased likelihood of current drinking compared to peers (10). This behavioral pattern reflects a dual crisis: while traditional drinking rituals persist, modern psychosocial stressors drive compensatory alcohol consumption as transient psychological analgesia.

These entrenched social norms present formidable challenges for healthcare professionals advocating for reduced alcohol consumption among youth.

## Intervention strategies by healthcare professionals

Chinese healthcare professionals employ a multifaceted, culturally sensitive approach to mitigate adolescent alcohol consumption, integrating public health initiatives, educational campaigns, and traditional practices. The following outlines the key strategies implemented (Table 1).

**Table 1:** Multifaceted intervention strategies for reducing adolescent alcohol consumption.

**Intervention approach**	**Key activities and components**	**Target audience and setting**
Individualized counseling and clinical interventions	One-on-one risk education (e.g., hepatic, cardiovascular effects); tailored strategies (limit-setting, trigger identification); referrals for dependency; connection to community support networks (e.g., mental health hotlines).	Adolescents presenting with alcohol-related health concerns; clinical settings.
National public health campaigns	Integration into the “Healthy China 2030” initiative; public education on dangers of excessive alcohol; community workshops, health fairs, and consultations; dissemination of educational materials; observance of events like World No Alcohol Day.	General public; communities; workplaces; nationwide.
School-based and family-centric digital interventions	Collaboration with schools for targeted sessions; family-oriented messaging; leveraging digital platforms (WeChat, Douyin) for infographics/videos; recommending monitoring apps and online support groups.	Adolescents in schools; families; digital environment.
Professional development and policy advocacy	Continuous education for HCPs (brief interventions, motivational interviewing, cultural competence); advocacy for stringent enforcement of laws restricting sales to minors.	Healthcare professionals; policy makers.

### Individualized counseling and clinical interventions

When adolescents present with alcohol-related health concerns, healthcare providers deliver one-on-one counseling to elucidate the associated risks, including hepatic dysfunction, cardiovascular complications, and adverse drug interactions (11–13). Tailored strategies, such as setting consumption limits or identifying behavioral triggers, are proposed while acknowledging the individual's sociocultural context. For cases of dependency, referrals to specialists or hospital-based programs are facilitated. Additionally, patients are connected to community support networks, including mental health hot lines, to ensure sustained intervention. Addressing stigmas and employing culturally appropriate communication are critical to fostering patient engagement.

### National public health campaigns

Healthcare professionals actively contribute to the “Healthy China 2030” initiative, a national strategy aimed at enhancing population health (14, 15). Within this framework, they spearhead public education efforts to highlight the dangers of excessive alcohol use, particularly its exacerbation of chronic conditions. Community workshops, health fairs, and consultations promote moderation or abstinence, while healthier alternatives to alcohol are advocated. Collaboration with social workers ensures a coordinated approach, and local health initiatives engage schools, workplaces, and community leaders to cultivate a culture of wellness. Educational materials, such as posters and pamphlets, are disseminated widely, and awareness campaigns are amplified during events like World No Alcohol Day. While the “Healthy China 200” campaign has achieved nationwide coverage, effectiveness varies significantly across regions. Health education efficacy is further moderated by sociocultural contexts. As evidenced by a multi-province survey of Chinese adolescents (*n* = 22,628), students with limited health literacy demonstrated 1.68-fold higher binge drinking prevalence than high-literacy peers (95% CI = 1.47–1.92), with parental education and urbanicity explaining part of this variance (16).

### School-based and family-centric interventions

Recognizing the vulnerability of adolescents, healthcare providers collaborate with educational institutions to deliver targeted sessions on alcohol-related risks. Family-oriented messaging is incorporated to harness familial influence in deterring early alcohol use. Digital platforms, including WeChat and Douyin (TikTok), are leveraged to disseminate infographics, videos, and testimonials advocating reduced consumption. Mobile applications that monitor intake or provide access to online support groups are recommended to align with the tech-savvy preferences of younger demographics (17). Conventional intervention approaches often fail to effectively engage adolescent populations. Given this demographic's heightened receptivity to digital innovations compared to other age groups, Internet-delivered strategies demonstrate superior potential for modifying youth alcohol-related attitudes and behaviors. To empirically validate this advantage, a Hong Kong research team pioneered an interactive web-based intervention targeting underage drinking reduction (18). Subsequently, they conducted a RCT (*n* = 7,792 adolescents) research to demonstrated that a 4-week web-based quiz game intervention achieved 21% immediate reduction (RR = 0.79, *P* = 0.003) and 14% sustained reduction (RR = 0.86, *P* = 0.048) in underage drinking prevalence at 1- and 3-month follow-ups, respectively, outperforming conventional health education (19).

### Professional development and policy advocacy

Healthcare professionals engage in continuous education to enhance their expertise in alcohol reduction and addiction management. Accredited online courses, expert-led workshops, and specialized certifications equip them with advanced skills in brief interventions, motivational interviewing, and culturally competent communication. Furthermore, they advocate for stringent enforcement of policies restricting alcohol sales to minors, utilizing these regulations as educational tools to reinforce public health messaging.

## The limited efficacy of interventions

The intervention practices outlined in this article—encompassing personal (counseling), community (seminars, events), and digital (social media and application-based) approaches—are presented in an idealized manner. However, empirical evidence suggests that their real-world implementation often yields suboptimal effectiveness. Longitudinal data reveal persistent challenges: approximately three decades ago, a marked escalation in alcohol consumption among adolescents was observed, accompanied by a significant surge in problem drinking rates notably prevalent among high school students in Japan—a developed Asian nation (20). Regrettably, current epidemiological data indicate that alcohol use continues to exhibit an upward trajectory in developing countries (21). In China, the prevalence of alcohol consumption among adults aged 15+ surged from 17.94% in 1991 to 34.3% during the 2010–2012 survey period, indicating a near-doubling of alcohol use rates over two decades (22, 23).

A 2021 survey reported that 82.5% of underage middle and high school students unimpeded alcohol purchases, reflects the lax implementation of existing protection laws (24). The weakness in enforcement can be attributed to several factors: first, fragmented regulatory oversight with responsibilities dispersed across multiple agencies (e.g., market regulation, commerce, and public security), leading to coordination challenges and accountability gaps; second, insufficient penalties for violations, which fail to deter retailers from selling alcohol to minors; third, limited resources for monitoring and enforcing compliance, particularly in vast rural areas and numerous small retail outlets. Aggressive advertising of alcohol campaigns permeate digital platforms and physical spaces, disproportionately influencing adolescents. The recent viral phrase “the body may not need alcohol, but the soul does” epitomizes youth resistance to abstinence-only messaging. A study about the trends of drinking behaviors among adolescents in Shanghai from 2004 to 2019 showed that the prevalence of ever drinking among adolescents in Shanghai over the observed period showed a declining trend by years, but no significant change in current drinking among adolescents (25). Indicated intervention approaches remain suboptimal for those with established drinking behaviors. These findings align with Taiwan's cognitive intervention model, where suppressing drinker self-schemas and rewiring outcome expectancies boosted refusal self-efficacy among abstinent teens, reducing 6-month drinking initiation (26). The majority clinical intervention studies are based on international researches. At present, there is a lack of intervention studies targeting drinking behaviors among adolescents in mainland China. More high-quality studies are needed to verify the effectiveness of these intervention strategies.

## Balancing cultural sensitivity and public health priorities

Alcohol in developing China represents a duality: a revered cultural symbol and a growing public health concern. Addressing this paradox necessitates a nuanced approach that respects tradition while advocating for evidence-based interventions. Healthcare professionals must employ tactful messaging to reconcile cultural practices with health promotion, fostering an equilibrium that encourages healthier lifestyle choices without alienating societal norms.

## Summary

Chinese healthcare professionals lead multifaceted efforts to reduce adolescent alcohol consumption through individualized counseling, national campaigns, educational outreach, and professional advocacy; However, interventions remain aspirational or rhetorical, with limited real-world implementation scant empirical evaluation of intervention efficacy exists to inform evidence-based policy making. particularly regarding their longitudinal impacts on behavioral trajectories. Future initiatives should prioritize three informed reconciliation strategies: ritual redesign, policy-culture integration and digital counter-marketing. In conclusion, adapting global best practices to China's unique cultural and institutional context, these efforts aim to enhance life expectancy, alleviate the burden of chronic diseases, and elevate the quality of life for future generations.
